# Antimicrobial activities of chicken β-defensin (4 and 10) peptides against pathogenic bacteria and fungi

**DOI:** 10.3389/fcimb.2015.00036

**Published:** 2015-04-17

**Authors:** Haitham A. Yacoub, Ahmed M. Elazzazy, Osama A. H. Abuzinadah, Ahmed M. Al-Hejin, Maged M. Mahmoud, Steve M. Harakeh

**Affiliations:** ^1^Biological Sciences Department, Faculty of Sciences, King Abdulaziz UniversityJeddah, Saudi Arabia; ^2^Genetic Engineering and Biotechnology Division, Cell Biology Department, National Research CentreGizza, Egypt; ^3^Biological Sciences Department, Faculty of Science, University of JeddahJeddah, Saudi Arabia; ^4^Division of Pharmaceutical and Drug Industries, Department of Chemistry of Natural and Microbial Products, National Research CentreGizza, Egypt; ^5^King Fahd Medical Research Center, King Abdulaziz UniversityJeddah, Saudi Arabia; ^6^Division of Human Genetics and Genome Research, Department of Molecular Genetics and Enzymology, National Research CentreGizza, Egypt; ^7^Special Infectious Agents Unit, King Fahd Medical Research Center, King Abdulaziz UniversityJeddah, Saudi Arabia

**Keywords:** antimicrobial activity, synthetic AvBD-4 and 10, bacteria, fungi, natural antibiotic

## Abstract

Host Defense Peptides (HDPs) are small cationic peptides found in several organisms. They play a vital role in innate immunity response and immunomodulatory stimulation. This investigation was designed to study the antimicrobial activities of β-defensin peptide-4 (sAvBD-4) and 10 (sAvBD-4) derived from chickens against pathogenic organisms including bacteria and fungi. Ten bacterial strains and three fungal species were used in investigation. The results showed that the sAvBD-10 displayed a higher bactericidal potency against all the tested bacterial strains than that of sAvBD-4. The exhibited bactericidal activity was significant against almost the different bacterial strains at different peptide concentrations except for that of *Pseudomonas aeruginosa* (*P. aeruginosa*) and *Streptococcus bovis* (*Str. bovis*) strains where a moderate effect was noted. Both peptides were effective in the inactivation of fungal species tested yielding a killing rate of up to 95%. The results revealed that the synthetic peptides were resistant to salt at a concentration of 50 mM NaCl. However, they lost antimicrobial potency when applied in the presence of high salt concentrations. Based on blood hemolysis studies, a little hemolytic effect was showed in the case of both peptides even when applied at high concentrations. The data obtained from this study indicated that synthetic avian peptides exhibit strong antibacterial and antifungal activity. In conclusion, future work and research should be tailored to a better understanding of the mechanisms of action of those peptides and their potential use in the pharmaceutical industry to help reduce the incidence and impact of infectious agent and be marketed as a naturally occurring antibiotic.

## Introduction

Host Defense Peptides (HDPs) are a diverse group of small and cationic peptides that are present in several organisms (Cuperus et al., 2013). Originally, they were called Antimicrobial Peptides (AMPs) due to their ability to inactivate and control bacteria *in vitro*. Several studies showed that these peptides have additional functions, mainly immunomodulatory ones (Cuperus et al., 2013). Therefore, they were named as “Host Defense Peptides (HDPs).” Recently, some reports indicated that HDPs are responsible for differentiation, activation and chemotaxis of leukocytes. They inhibited Lipopolysaccharide (LPS), enhance phagocytosis, DNA uptake and wound healing (Zanetti, 2005; Semple and Dorin, 2012).

HDPs were first discovered in the 1970's by extraction from tissues of avian species (Evans et al., 1994). Based on new advances in the field of bioinformatics and available sequence data, a number of new HDPs have been identified. The first avian HDPs were confirmed in the mid 1990's, and included five defensins isolated from the leukocytes of some avian species like chickens and turkeys (Evans et al., 1994). Recently, the complete defensin and cathelicidin genes clusters have been sequenced for chickens. Due to the interest in their role as potential therapeutically agents, there is a remarkable amount of information based on various studies using other avian species (Cuperus et al., 2013). Currently, there is a lot of research on the role of these peptides as templates for possible novel anti-microbial agents (Cuperus et al., 2013). Accordingly, they have been sought as possible alternatives to antibiotics which lost their shine due to the increasing resistance that is exhibited against a wide range of bacteria which is a major public health issue especially during the last decade (Cuperus et al., 2013). Defensins peptides are cysteine-rich, cationic in nature and composed of three conserved disulfide bridges, a β-sheet and both hydrophobic and cationic amino acids (Ganz, 2003; Selsted and Ouellette, 2005). The defensin peptides are classified into three main groups: α−, β−, and θ−defensins (Yang et al., 2004; Selsted and Ouellette, 2005). Alpha-defensins are found in mammals and form disulfide bridges between Cys1–Cys6, Cys2–Cys4, and Cys3–Cys5 (Lehrer and Ganz, 2002; Yang et al., 2004). Theta-defensins are present in all vertebrates and form disulfide bonds between Cys1–Cys5, Cys2–Cys4, and Cys3–Cys6 (Lehrer and Ganz, 2002; Klotman and Chang, 2006). Based on extensive search and based on chicken genome analysis, β-defensins were the first to be confirmed which derived from avians species. Using the chicken genome, they were first to be confirmed in avian species (Lehrer and Ganz, 2002; Klotman and Chang, 2006). The underlying mechanism by which AMPs exert their effect is through their interaction with the negatively charged phosolipid bilayer found in the cell membrane under hydrophobic conditions, thus resulting in disruption and killing of the pathogen (Higgs et al., 2005; Lynn et al., 2007). It has been reported that avian β-defensin are more efficient against Gram-positive bacteria than Gram-negative bacteria due to their structural conformation (Cuperus et al., 2013). The mode of action of theses peptides in bacterial killing was reported in some studies due to its net charge. Some studies revealed that the duck β−defensin-12 has the lowest net charge and lower antibacterial activity as compared to duck β−defensin 4 and 7 peptides (Powers and Hancock, 2003; Ganz, 2004; Brogden, 2005).

This study was designed to evaluate the antimicrobial activities of synthetic chicken β-defensin peptides (sAvBD-4 and 10) against both pathogenic Gram-negative and Gram-positive bacteria and fungi. The kinetics were also investigated and the effect of salinity on their activity was delineated.

## Material and methods

### Bacterial species

Bacterial species used included both Gram-negative and Gram-positive strains. The five Gram-positive bacterial strains used were: *Micrococcus luteus* ATCC 49732 (*M. Luteus*), *Enterococcus faecalis* ATCC 29212 (*Ent. faecalis*), *Streptococcus bovis* ATCC 49147 (*Str. bovis*), *Staphylococcus epidermidis* ATCC 12228 (*Staph. epidermidis*) and Methicillin-Resistance *Staphylococcus aureus* ATCC 43330 (MRSA). Five strains of Gram-negative bacteria included: *Pseudomonas aeruginosa* ATCC 27853 (*P. aeruginosa*), *Escherichia coli* ATCC 25922 (*E. coli*), *Salmonella typhimurium* ATCC 14028 (*Salm. typhimurium*), *Klebsiella pneumonia* ATCC 700603(*Kleb. pneumonia*), *Shigella sonnei* ATCC 25931(*Sh. sonnei*). The bacterial strains were cultured on tryptone soy agar and incubated for 24 h at 37°C and stored in slants at 4°C.

### Fungal species

Three types of fungal species were used; *Candida albicans* ATCC 10231 (*C. albicans*), *Aspergillus flavus* (*Asp. flavus*), and *Aspergillus niger* (*Asp. niger*) isolates (obtained from the Department of Biological Sciences, King Abdulaziz University, Jeddah, Saudi Arabia. Filamentous fungi and Candida were cultured in Sabouraud dextrose agar and incubated at 25–28°C for 72 h for multicellular fungi and h at 30°C for 24 in the case of Candida and stored in slants at 4°C.

### Chicken β-defensins peptides synthesis

The mature peptide of chicken β-defensin (AvBD-4 and 10) were custom synthesized and purified to 80% level using high-performance liquid chromatography (HPLC) by GL Biochem Ltd. (Shanghai, China) (http://www.glschina.com/en/profile.htm). The full sequence of amino acids of chicken β-defensin (AvBD-4 and 10) are listed in Table 1.

**Table 1:** **Amino acid sequence of synthetic AvBDs**.

	**Amino acid sequence**	**AA**
AvBD-4	AC-MKILCFFIVLLFVAVHGAVGFSRSPRYHM QCGYRGTFCTPGKCPHGNAYLGLCRPKYSCCRWL	64
AvBD-10	AC-MKILCLLFAVLLFLFQAAPGSADPLFPDTVACRT QGNFCRAGACPPTFTISGQCHGGLLNCCAKIPAQ	69

### Antimicrobial activity assay

MIC assays for the peptides were performed by two-fold broth dilution method with Mueller Hinton II broth according to the procedures as suggested by the CLSI (Clinical and Laboratory Standards Institute) (Wayne, 2009, 2012). In summary, Bacteria and Candida were grew to reach out the exponential phase. Cells were then centrifuged at 2000 × g for 15 min. The resulting pellets were washed and resuspended in 10 mM sodium phosphate buffer saline solution (pH 7.0). Two-fold serial dilutions of the sAvBD-4 and 10 were prepared in appropriate culture medium in 96-microwell plates. 100 μL inoculum from the culture with a bacterial density of 106 CFU/ml were added to each individual well containing 100 μL of either M-H alone, or M-H containing two-fold diluted peptide to give a final concentration of the peptide ranging from 0 to 200 μM. MIC values were recorded as the minimum concentration that showed no visible growth after overnight incubation at 37°C. The cell survival percentage was determined by measuring the absorbance at λ = 570 nm with a microplate reader. Cell survival % = [(mean optical density of the sample − blank)/(mean optical density of the control ((no treatment) − blank)] × 100.

The minimum bactericidal concentration (MBC) or the minimum fungicidal concentration (MFC) were evaluated by subculture of the contents of the first two clear wells obtained in the MIC assay onto minimal M-H agar plates. The lowest peptide concentration yielding more than 99% of either bacterial or fungal growth inhibition was noted as MBC or MFC.

Two multicellular fungi (*Asp. niger* and *Asp. flavus*) were used to evaluate the anti-fungal activity of those two peptides. Overnight cultures of the fungi were prepared by inoculating 100 ml of Sabouraud dextrose broth with a 105 spores/ml concentration. Tetracycline used as a reference in the case of both Gram-negative and positive bacteria. On the other hand, Ketoconazole was use as a reference in the case of the fungus *C. albicans*, *Asp. Niger*, and *Asp. flavus*. The both reference antibiotics were used at concentration of (30 μg/ml).

### Kinetics of inactivation

In order to study the kinetics of bacterial inactivation for both of the test peptides, three organisms were used: MRSA, and *E. coli* (1 × 10^8^ CFU/ml) and *C. albicans* (1 × 10^8^ CFU/ml). The concentration used was two times of that of the MIC. Overnight bacterial cultures were prepared. The cultures were spun down and resuspended in fresh M-H medium at a concentration of 1 × 10^8^ CFU/ml. sAvBD-4 and 10 were then added to the bacterial suspension, at a concentration equivalent to two times that of the MIC. The mixture was incubated under 35°C. Ten-microliter aliquots were removed with a sterile calibrated loop at (0, 15, 30, 60, 120, and 180 min) and uniformly seeded on M-H medium (Ma et al., 2011). The plates were incubated at 35°C for 24–48 h. After the incubation period, the number of viable cells was counted and expressed in CFU/ml. The results were analyzed and represented graphically, a microbial death curve as a function of time.

### Salinity test

*E. coli* was used as a test model to evaluate the effects of ionic strength on the antibacterial activity of the two peptides. *E. coli* was subcultured at 37°C to the mid-log phase, and suspended to 10^6^ CFU/ml in MH. A suspension of *E. coli* (1 ml) was incubated with different concentrations of peptides (0–200 μg/mL), with different concentrations of NaCl (0, 20, 50, 150 mM) in 10 mM sodium phosphate buffer, pH 7.4. The tested bacteria was cultured at 37°C for 2 h before 1000 times of dilution followed by plating. Survived bacteria were counted (Ma et al., 2011; Wang et al., 2011; Baricelli et al., 2015).

### Hemolysis test

The hemolytic activities of the synthetic defensin were investigated according to what has been reported in the literature (Shin et al., 2001; Yu et al., 2001). Briefly, fresh chicken blood was collected from King Abdulaziz University farm, Jeddah, KSA. The blood was spun down for erythrocytes harvesting by centrifugation (3000 rpm, 10 min, at 20°C). The resulting erythrocytes were then washed twice with sterile PBS at a concentration of 0.5% vol/vol and were used for the assay, by dispensing 90 μl into each well of the 96-well plates. Ten microliters of different peptides concentrations were added to the cells and incubated at 37°C for 2 h. After incubation, the microtiter was spun down at 800 × g for 10 min. The supernatants were withdrawn and transferred to a new 96-well plate and checked for released hemoglobin as measured spectrophotometerically at 405 nm. For (0 hemolysis) as well as (100% hemolysis) controls, cells were resuspended in PBS only and in 1% Triton X-100, respectively (Ma et al., 2013).

### Statistical analysis

Data were entered using IBM SPSS Statistics 20, and was analyzed by Kaplan–Meier analysis. A *P* level of < 0.05 was considered to significant.

## Results

**Figure 1:**
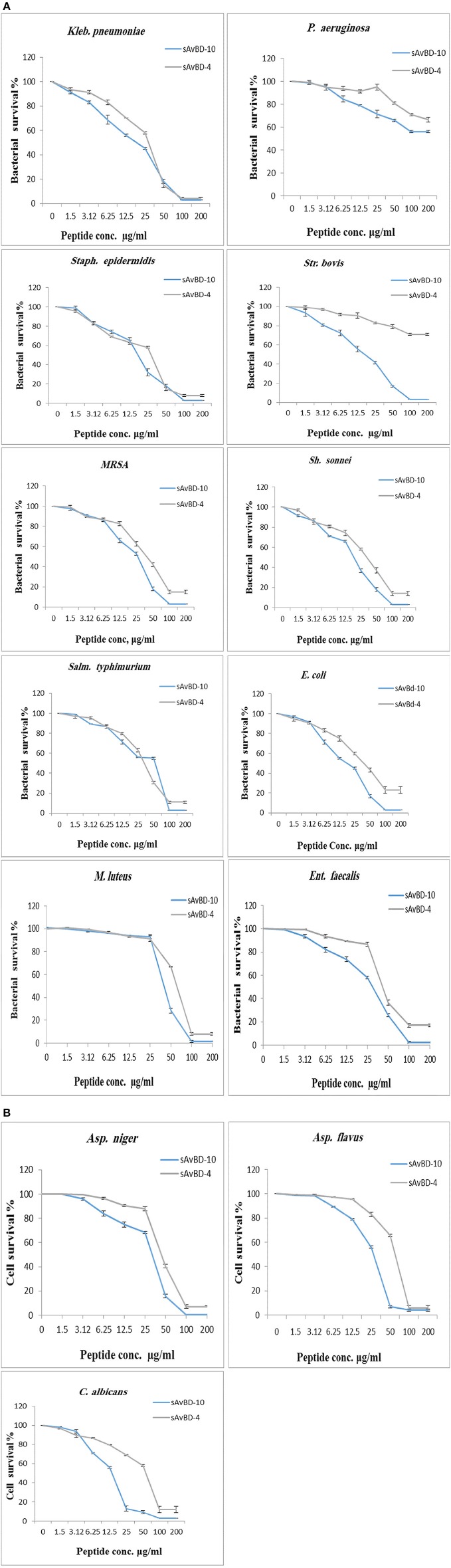
**Antimicrobial activities of synthetic chicken β-defensin-4 and 10-derived peptide (sAvBD) against (A) bacteria and (B) Fungal species**. All assays were performed in three independent experiments and each point is the mean ± SE, (*P* < 0.002).

The chicken β-defensin (sAvBD-4 and 10) used in this study were analogs of the natural peptides. These were custom made having the linear N-terminal acetylated as is the case with naturally occurring mature chicken β-defensin peptides. These custom-made peptides were evaluated for their anti-bacterial and anti-fungal activities against 10 bacterial strains and three fungal species. There was a variation in the response of the bacteria to the tested peptides with sAvBD10 showing a better efficacy on average against all the bacteria tested. Statistical analyses showed that the difference was significant at the 95% as shown in (Table 2, Figures 1A,B). The results showed that sAvBD-4 inhibited the growth of both Gram-negative and positive bacteria with MIC concentrations as follows: 25 μg/ml [(*Staph. epidermidis, Kleb. pneumonia, Sh. sonnei, C. albicans*), 50 μg /ml (MRSA, *M. luteus, Salm. typhimurium, E. coli, Asp. flavus*) and 100 μg/ml for (*Str. bovis, Ent. faecalis, Asp. niger*)]. However, sAvBD-10 was more efficient in achieving bacterial inactivation at the following MIC concentrations: 25 μg/ml (*M. luteus, Kleb. pneumonia, C. albicans*, and *Asp. flavus*) and 50 μg/ml (*Str. bovis, Ent. faecalis*, MRSA, *Salm. typhimurium, E. coli, Staph. epidermidis, Sh. sonnei, Asp. niger*, and *P. aeruginosa*). The MBC levels were also determined and found to be two-fold higher than those of the corresponding MIC values (MBC range, 50–200 μg/ml) (Table 2). At those lower concentrations, sAvBD-10 had a significantly better antimicrobial activity as compared to sAvBD-4 against all the bacteria tested (*P* < 0.002). However, at higher concentrations of 100 μg/ml, both peptides showed no significant difference in their bactericidal efficacy (Table 2).

**Table 2:** **The antimicrobial activities of synthetic chicken β-defensin-4 and 10-derived peptide**.

**Organisms species**	**sAvBD-4 peptide**		**sAvBD-10 peptide**		**Tetracycline**	
**Bacteria**	**MIC (μg/ml)**	**MBC (μg/ml)**	**MIC (μg/ml)**	**MBC (μg/ml)**	**MIC (μg/ml)**	**MBC (μg/ml)**
*Str. bovis ATCC (49147)*	<100±0.0	200±0.0	50±0.0	100±0.58	10±3.46	20±0.0
*Staph. epidermidis ATCC (12228)*	25±3.46	100±0.58	50±0.0	100±0.58	10±1.73	10±0.0
*Staph. aureus MRSA ATCC (43330)*	50±0.0	100±1.0	50±1.3	100±1.0	20±0.0	<20±1.73
*Ent. faecalis ATCC (29212)*	100±0.0	200±0.58	50±0.0	100±1.73	10±0.0	20±0.0
*M. luteus ATCC (49732)*	50±3.0	50±1.58	25±0.0	50±1.73	5±0.0	10±0.0
*E. coli ATCC (25922)*	50±1.3	100±0.00	50±1.73	100±3.46	10±0.0	20±1.73
*P. aeruginosa ATCC (27853)*	–	–	50±0.0	200±3.46	10±1.73	20±1.73
*Salm. typhimurium ATCC (14028)*	50±0.58	100±3.46	50±0.58	100±3.46	10±0.0	20±0.0
*Kleb. pneumonia ATCC (700603)*	25±0.0	50±0.58	25±0.0	100±1.73	5±0.0	20±0.0
*Sh. sonnei ATCC (25931)*	25±3.46	100±3.46	50±1.73	100±0.58	5±0.0	10±1.0
**Fungi**		**MFC**		**MFC**	**Ketoconazole**	
*C. albicans ATCC (10231)*	25±1.0	100±0.58	25±0.0	50±0.58	3±0.0	3±0.0
*Asp. Flavus*	50±1.0	100±3.46	25±0.0	50±0.58	10±0.0	25±0.0
*Asp. Niger*	100±1.73	–	50±3.0	100±1.3	10±0.0	10±0.0

*Determination of MICs (μg/ml) and MBCs (μg/ml), or MFCs (μg/ml) for each peptide and bacteria was performed at least three-times in doublets. sAvBD-4, synthetic chicken β-defensin-10; sAvBD-10, synthetic chicken β-defensin-10; MIC, minimal inhibition concentration; MBC, minimal bactericidal concentration; MFC, minimal fungicidal concentration*.

There was a dose dependent decline in antifungal inhibition and inactivation by both peptides (Table 2). Again, sAvBD-10 showed a significantly better anti-fungal activity as compared to sAvBD-4 as far as both MIC and MFC are concerned. In the case of sAvBD-10 (Figure 1B), the MIC varied among the three fungi with 25 μg/ml needed for both *C. albicans* and *Asp. flavus* and 50 μg/ml for *Asp. niger*. There was a significant difference in the MIC reported regarding sAvBD-4 among the three fungal species tested (Table 2, Figure 1B) with highest MIC observed in the case of *Asp. niger*.

The data showed that the MFC activity of sAvBD-10 was 25 μg/ml in the case of *C. albicans* and *Asp. flavus* and 50 μg/ml for *Asp. niger*. A much higher concentration was needed concerning sAvBD-4 (Table 2, Figure 1B). A better bactericidal activity was noted in the case of tetracycline as compared to the tested peptides. This, also, applies in the case of ketoconazole.

### Kinetics of inactivation

**Figure 2:**
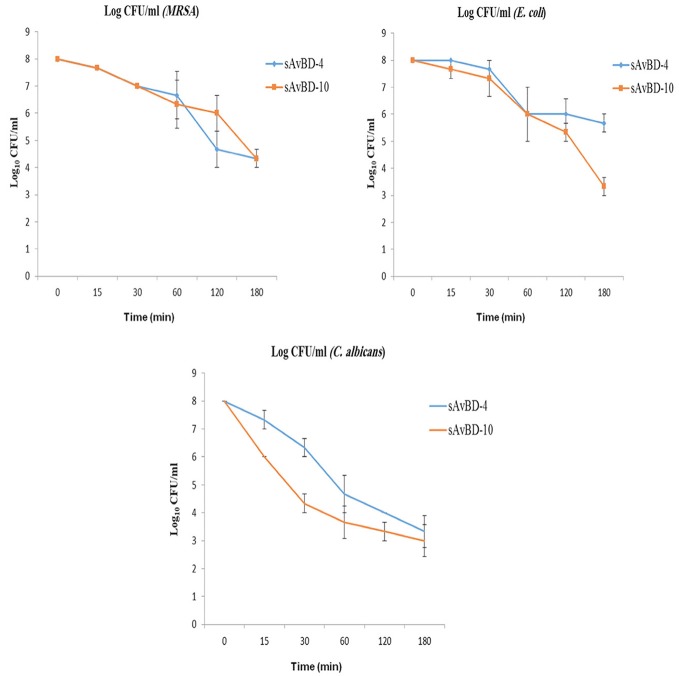
**The kinetic inactivation of synthetic chicken β-defensin-4 and 10-derived peptide (sAvBD) against MRSA, *E. coli* and *C. albicans* species**. All assays were performed in three independent experiments and each point is the mean ± SE.

In the kinetics study, MRSA, *E. coli*, and *C. albicans* were used (Figure 2). Both peptides showed similar efficacy against MRSA during the first hour of application. However, faster decline was prominent in the case of sAvBD-4 for the next 2 h. This was not the case in term of *E. coli* where the two peptides started the same for the first hour and sAvBD-10 produced higher bactericidal efficacy for the remaining 2 h. As far as the kinetics of killing of *C. albicans* is concerned, there was a consistent better efficacy of sAvBD-10 over sAvBD-4.

### Salinity test

**Figure 3:**
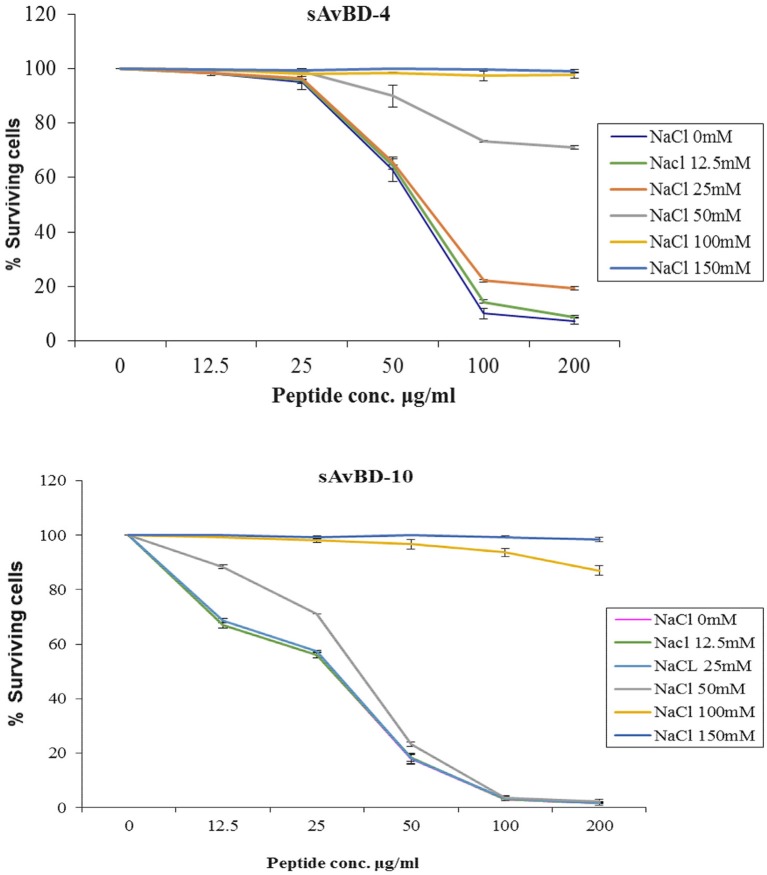
**Effects of salinity on the antibacterial activity of synthetic chicken β-defensin-4 and 10-derived peptide (sAvBD) against *E. coli***. All assays were performed in three independent experiments and each point is the mean ± SE, (*P* < 0.001).

The effect of salinity on the antibacterial efficacy of both peptides was evaluated using various salt concentrations ranging from 0 to 150 mM (Figure 3). The results revealed that the synthetic peptide's efficacy was not reduced in the presence of salt concentrations ranging from 0 to 50 mM. However, such an effect was significantly compromised in a dose dependent manner when higher concentrations of salt were present (Figure 3) (*P* < 0.001).

### Hemolytic activity of synthetic peptide

**Figure 4:**
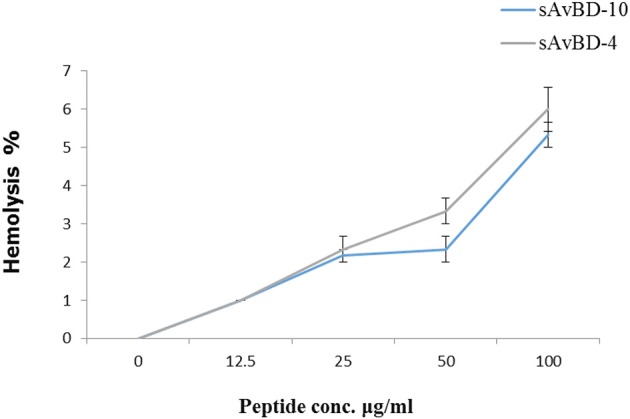
**Hemolytic activities of synthetic chicken β-defensin-4 and 10-derived peptides (sAvBD)**. All assays were performed in three independent experiments and each point is the mean ± SE.

The hemolytic activities of the two peptides were evaluated using freshly isolated chicken erythrocytes. The hemolysis was done using spectrophotometric measurements at a wavelength of 405 nm (Figure 4). The results showed that the peptides did not produce significant hemolytic effects.

## Discussion

Around 1000 HDP has been discovered so far. The β-defensins, cathelicidens and liver antimicrobial peptide-2 are three of those HDPs that originated from chickens. In this investigation, the antimicrobial potential of two synthetic peptides (AvBD-4 and 10) was evaluated against 10 Gram-positive and -negative bacterial strains, as well as unicellular and multicellular fungi. This work was initiated to better understand the efficacy of those novel peptides against bacteria and fungi and their possible application in conjunction either with already existing antimicrobial agents or with possible substitutes for them. The uncontrolled use, misuse, and abuse of commonly administered antimicrobial agents has resulted in the horizontal gene transfer among microorganisms and led to the stimulation of the evolutionary potential of the bacteria to develop some kind of resistance against the antimicrobial agents and consequently resulted in the emergence of multi-drug resistant bacteria. Such a resistance to antimicrobials is a worldwide problem in the treatment of infectious diseases especially in patients who are immunocompromised patients with hospital-acquired infections. For this reason, alternative agents have been sought to deal with the issue of multi-drug resistance. The AMPs are naturally occurring products, which have undergone a long-term evolution in nature. These peptides may have a promising potential as possible substitutes for antimicrobial agents or to potentiate the efficacy of already existing drugs used for the treatment of infectious agents.

The findings of this study revealed that the synthetic AvBD-4 and 10 displayed a potent efficacy against a broad-spectrum of bacteria. The current results were compatible with those of other studies on the antimicrobial activity of other known AvBDs (Evans et al., 1994, 1995; Harwig et al., 1994; Thouzeau et al., 2003; Lynn et al., 2004; Higgs et al., 2005; van Dijk et al., 2008; Ma et al., 2009a,b, 2011, 2012a,b,c; Soman et al., 2010; Wang et al., 2010; Zhang et al., 2011, 2012). For instance, geese derived synthetic peptides showed an effect against both Gram-positive and -negative bacteria, like *S. aureus* and *E. coli*, thus consistent with the findings on synthetic β-defensins potency in their natural forms as reported elsewhere (Sass et al., 2010; Ma et al., 2013). The results of a study on recombinant AvBD2 derived from ducks exhibited a strong bactericidal potential against *B. cereus*, *S. aureus*, and *P. multocid*, and weak bactericidal activity against *E. coli* and *S. choleraesuis* like the results obtained using defensins (Evans et al., 1994; Harwig et al., 1994; Yu et al., 2001; Thouzeau et al., 2003; Sugiarto and Yu, 2006), were chemically synthesized (Higgs et al., 2005; van Dijk et al., 2007) or were produced by recombinant expression (Milona et al., 2007; van Dijk et al., 2007; Ma et al., 2008). In another study using Recombinant HBD2 showed that it inhibited three Gram-negative bacteria known for their resistant to antimicrobial agents (*S. marcescen, P. aeruginosa, A. baumannii*) that are opportunistic nosocomial pathogens present among immunocompromised individuals (Baricelli et al., 2015).

Interestingly, besides the broad spectrum of antibacterial effect, the synthetic AvBD-4 and 10 exhibited a fungicidal activity against both unicellular and multicellular fungi. More than 90% fungicidal activity was reported in this study. It was interesting to note that avian derived peptides have an anti-fungal potential. The results showed for the first time that those avian peptides had inhibitory and cidal effects against fungi. Also, it was reported that defensin secreted by filamentous fungi displayed strong inhibitory potential against human and plant infectious agents (Lacadena et al., 1995; Meyer, 2002, 2008; Marx, 2004; Galgóczy et al., 2010).

The differences in the inactivation kinetics between the two peptides may be structure related and/or dependent on the type bacteria used. Studies demonstrated that pheasant cathelicidin-1 (Pc-CATH1) inhibited *E. coli* growth during the first hour of application. However, it took 6 h for the bacterial growth to be resumed (Wang et al., 2011). The killing kinetics for synthetic sAvBD-10 was more effective than sAvBD-4 in eliminating fungal species than bacteria as indicated in this investigation.

Salt resistance is a defiance that is highly pertinent for HDPs in order for them to exert their full potential under physiological conditions. The results showed that the NaCl levels as those found under physiological conditions found in the human body did not compromise the efficacy of the tested peptides. Similar to those present in the Similar to other defensins (Sang et al., 2005; Ma et al., 2011). The efficacies of those peptides were compromised in the presence of high salt concentrations. This is consistent with the findings reported by others (Soman et al., 2010) and those of others using ostrich (Ma et al., 2013; Lu et al., 2014). The results revealed that the effect of ionic strength on the antimicrobial impact of investigated peptides were compatible with other reports on most tested defensins and other AMPs (Porter et al., 1997; Bals et al., 1998; Zucht et al., 1998; Veldhuizen et al., 2008).

Furthermore, the antimicrobial potency of synthetic AvBD-4 and 10, these synthetic peptides had low hemolytic effect and low toxicity at higher concentrations. These results are in agreement with other studies on other avian β-defensin and also for mammals defensin (Milona et al., 2007; Veldhuizen et al., 2008; Ma et al., 2011, 2012a,c). Some published studies indicated that the little toxic effect of defensin peptide on animal cells might be due to the partly higher cholesterol levels and a lack of negatively charged phospholipids in the outer leaflet of animal membranes, which inhibit the binding of many AMPs (Matsuzaki et al., 1995; Ishitsuka et al., 2006). This in agreement with the data obtained by other scientists using geese and duck related defensins (Ma et al., 2013; Lu et al., 2014).

The mechanism of action by which those peptides these peptides exert their effects is not well-understood. It is likely to be due to interaction of the peptides which are positively charged with specific structures like the phospholipid membranes found on the negatively charged bacterial membrane. The result of the interaction of the peptides with the phospholipid membranes, will lead to an increase in the pore formation of the membrane which result in increased permeability leading to the demise of the bacterial cell (Zasloff, 2002; van Dijk et al., 2008; Derache et al., 2009; Ma et al., 2013; Lu et al., 2014).

## Conclusion

The antimicrobial activity of synthetic chicken β-defensin-4 and 10 were studied against wide spectrum of infectious pathogens, including bacterial and fungal species. These peptides have exhibited significant antimicrobial activities against a broad spectrum of Gram-positive and Gram-negative bacteria of concern to the public health and have been involved in many outbreaks. Interestingly, besides their broad spectrum of antibacterial activities, synthetic AvBD (4 and 10) have shown fungicidal potential against *C. albicans* and *Asp. flavus*. The results showed that the synthetic AvBD (4 and 10) were more efficacious as an anti-fungal agents causing more than 95% reduction in fungi as compared to the control. Both of those synthetic peptides were resistant to salt concentrations of 50 mM NaCl, but lost their antimicrobial potential in a milieu containing salt concentration of 100 and 150 mM NaCl. Based on the data, it would be concluded that synthetic β-defensin peptides have a potent antimicrobial activities against a broad spectrum of pathogens and would merit the need for more studies to investigate their full potential in the treatment of disease in both humans and animals. They are likely to be a possible alternative to antibiotics and serve as natural antimicrobial properties with the hope that they do not produce bacteria that are resistant due to their naturally occurring properties.

## Author contributions

HY and AE collection and assembly of the data, manuscript writing, and data analysis; HY, AE, MM, and SH discussion, manuscript revision; AA, MM, and OA data analysis and discussion; HY and AE concept and design, data analysis, manuscript revision, and final approval of the manuscript.

### Conflict of interest statement

The authors declare that the research was conducted in the absence of any commercial or financial relationships that could be construed as a potential conflict of interest.
